# Reply to “A resurrection of the Haber-Weiss reaction”

**DOI:** 10.1038/s41467-021-27824-1

**Published:** 2022-01-19

**Authors:** Yumeng Zhao, Meng Sun, Menachem Elimelech

**Affiliations:** 1 State Key Laboratory of Urban Water Resource and Environment, Harbin Institute of Technology, 150090 Harbin, China; 2 Department of Chemical and Environmental Engineering, Yale University, New Haven, CT 06520-8286 USA; 3 Center for Water and Ecology, State Key Joint Laboratory of Environment Simulation and Pollution Control, School of Environment, Tsinghua University, 100084 Beijing, China

**Keywords:** Pollution remediation, Environmental impact, Electrocatalysis

**replying to** Willem H. Koppenol *Nature Communications* 10.1038/s41467-021-27823-2 (2022)

In our recent published paper^1^, we presented an entirely new design of a Janus electrocatalytic membrane and demonstrated its efficient water decontamination performance. The Janus electrocatalytic membrane demonstrated in situ singlet oxygen (^1^O_2_) formation inside the membrane porous structure, enabling enhanced removal of contaminants from water in a single-pass electrofiltration at very low energy consumption and without the addition of chemical precursors. The enhanced water decontamination performance was ascribed to the electrocatalytic membrane design. The Janus membrane integrates both the anodic and cathodic reactions within the membrane porous structure during flow-through filtration. This unique membrane structure induces spatial confinement within the membrane inner pores and enhances convective mass transport of reactants. In contrast to traditional electrofiltration designs with membranes functioning either as cathode or anode, using a Janus membrane makes full use of electrical energy, thus promoting energy efficiency.

In the accompanying Comment, Dr. Koppenol raised concerns regarding the ^1^O_2_ formation pathways in the Janus electrocatalytic membrane. In our work, we proposed potential ^1^O_2_ formation pathways in the membrane. Due to limitations of in situ characterization techniques inside a porous membrane, we were not able to provide the exact reaction pathways for ^1^O_2_ formation without solid experimental evidence. We appreciate Dr. Koppenol’s effort in proposing an alternative hypothesis for the ^1^O_2_ formation pathway in the Janus electrocatalytic membrane, which involves O_2_^•−^ generation at the cathode followed by anodic O_2_^•−^ oxidation for ^1^O_2_ formation.

The second concern raised by Dr. Koppenol is whether ^•^OH exists in the Janus electrocatalytic membrane. As stated in our manuscript, the Pt anode is inherently an “active” anode. With its low oxygen evolution overpotential, the “active” anode readily transforms the generated physisorbed ^•^OH into a higher oxide. This analysis has been proven by both theoretical and experimental studies^2,3^. If ^•^OH is generated in the Janus electrocatalytic membrane, it would be readily converted to the higher oxide PtO. Therefore, free ^•^OH would not be detected in the membrane.

The third concern raised by Dr. Koppenol is the suitability of electron paramagnetic resonance (EPR) measurement using 2,2,6,6-tetramethylpiperidine (TEMP) as the trapping agent for ^1^O_2_ detection. The general consensus is that TEMP is able to react with ^1^O_2_, forming 2,2,6,6-tetramethyl-1-piperidinyloxy (TEMPO). The TEMPO product exhibits a characteristic 1:1:1 triplet signal in the EPR spectrum, serving as an indicator for ^1^O_2_ existence. This method has been widely applied in the field of environmental science as an indicator method for ^1^O_2_ detection^4,5^. Nevertheless, the EPR-TEMP detection method is currently being discussed^6^, as it cannot exclusively indicate ^1^O_2_ formation. This is because TEMPO may possibly be generated by another route, wherein TEMP^•+^ intermediate radical is first formed, then undergoes deprotonation and reaction with molecular oxygen^6^.

As we stated above, in situ characterization techniques of stepwise transformations of the intermediates inside the porous membrane structure are challenging. Hence, at present, we cannot confirm the TEMP transformation pathway inside the membrane. Given this situation, we employed other detection agents—furfuryl alcohol (FFA) and sulfamethoxazole (SMX)—to confirm ^1^O_2_ formation. The oxidized products of FFA and SMX by ^1^O_2_ (Figs. S11 and S17 in the Supporting Information of our article^1^) were in agreement with previously reported results^7,8^. Overall, we adopted different detection methods to collectively confirm ^1^O_2_ formation in the membrane.

Regarding the ROS terminology, Dr. Koppenol states that neither O_2_^•−^ nor H_2_O_2_ are reactive. We clarify that although H_2_O_2_ and O_2_^•−^ are not highly reactive species as ^•^OH, they are reactive with contaminants in water. H_2_O_2_ has been demonstrated as an effective disinfectant for inactivating bacteria and mitigating membrane fouling in water treatment^9^. In addition, the use of the term “ROS” or “reactive oxygen species” for describing H_2_O_2_, O_2_^•−^, ^•^OH, and ^1^O_2_ is widely known in the fields of chemistry and environmental science^10^.

The current Janus electrocatalytic membrane module has limitations in determining reaction pathways (i.e., ROS formation and pollutant degradation) occurring inside the membrane. These limitations are also found in conventional electrofiltration modules, where flat-sheet membranes function only as cathode or anode. We attribute these limitations to two main causes. First, the membrane electrode and the counter electrode coexist in the same chamber of the membrane module. Reactants and intermediates produced by the membrane electrode will flow to the counter electrode, inducing additional electrochemical reactions. Hence, analyzing the reactions developed exclusively by the electrocatalytic membrane could be interfered by the counter electrode. Second, unlike a heterogeneous batch reaction system, real-time, on-site monitoring of molecules in nanoscale membrane pores during electrofiltration is challenging. Due to spatial and temporal restrictions, advanced detection techniques for elucidating the dynamic and instantaneous transformation of reactants (e.g., stopped-flow spectrophotometry^11^) cannot be deployed in systems with confined and pressure-driven liquid transport.

Refining membrane module design is critical for clarifying the reaction mechanisms in electrocatalytic membranes. To scrutinize the two half-cell reactions independently, isolating the membrane electrode and the counter electrode (or the cathodic and anodic regions of the Janus membrane) during electrofiltration is necessary. Selective barriers, such as vertically aligned single-walled carbon nanotube membranes^12^, could be inserted between the two electrodes/regions. Such barriers allow electron transfer while blocking other substances (i.e., H_2_O, ROS, and organic pollutants). More importantly, exploiting materials and configurations of membrane modules that are integrated with time-resolved detection devices is of practical significance. The development of microfluidic chips compatible with advanced sensing techniques, such as luminescence^13^ and ion beam pulse^14^, is beneficial for in situ observation of phenomena (e.g., catalytic reaction, ROS generation, and ion solvation) occurring inside membrane pores.

In conclusion, we thank Dr. Koppenol for raising these issues. The answer to the main issue raised, i.e., unraveling ^1^O_2_ formation pathways, is very challenging to fully clarify by experiments at present. Like any newly developed materials and technologies, the Janus electrocatalytic membrane shows unique advantages, but also presents challenges and requires more study to fully understand its detailed molecular mechanisms. Importantly, compared with conventional electro-active membranes, the Janus electrocatalytic membrane is a sustainable and energy-efficient method for water purification. In addition, the Janus electrocatalytic membrane extends electro-active membrane functionalities beyond water purification, with potential applications in resource recovery and environmental sensing^15^. We hope the present work and the discussion with Dr. Koppenol can inspire more research to promote the development of the Janus electrocatalytic membrane and related in situ detection techniques.

## Data Availability

No new data were generated for this reply.
